# Oxygen limitation induces acid tolerance and impacts simulated gastro-intestinal transit in *Listeria monocytogenes* J0161

**DOI:** 10.1186/s13099-015-0058-0

**Published:** 2015-04-10

**Authors:** Danny Sewell, Stuart CH Allen, Carol A Phillips

**Affiliations:** School of Health, University of Northampton, Northampton, UK

**Keywords:** *Listeria monocytogenes*, Gastro-intestinal transit, Acid tolerance response, Anaerobiosis, Adaptation

## Abstract

**ᅟ:**

*Listeria monocytogenes* is a food-borne pathogen and the causative agent of listeriosis, a severe infection to those with a pre-disposition. Infections often arise through consumption of contaminated foods, where high intrinsic resistance to food processing practises permit survival and growth. Several practises, including refrigeration, acidification and oxygen limitation are ineffective in controlling *L. monocytogenes*, therefore foods which do not undergo thermal processing, e.g. ready-to-eat products, are considered high risk.

While the responses to several food processing practises have been investigated, there are few reports on the responses of *L. monocytogenes* to oxygen limitation. Therefore the aim of this study was to investigate the effects of oxygen limitation on stress response andsurvival capacity during simulated gastro-intestinal transit.

**Findings:**

Anaerobiosis induced an acid tolerance response, causing cells to be more resistant to organic and inorganic acids than aerobically grown counterparts (*p* < 0.05).

Using a gastro-intestinal transit model it was found that anaerobic growth induced an acid tolerance response which enhanced resistance to pH 2.5 simulated gastric juice (SGJ) compared to aerobically grown cells (*p* < 0.05). This response was most pronounced in exponential phase cells. However, exposure of stationary phase cells to pH 3.5 SGJ enhanced bile tolerance, suggesting a link between acid and bile tolerance.

**Conclusions:**

The responses of *L. monocytogenes* to oxygen limitation are not extensively studied. These findings provide an initial insight into the effects of anaerobiosis on stress response and survival potential in *L. monocytogenes*. While it appears anaerobiosis may impact these, further work is required to confirm these findings are not strain specific.

## Findings

*Listeria monocytogenes* is a Gram positive food-borne pathogen associated with human disease/illness, ranging from mild gastroenteritis to potentially fatal invasive listeriosis [1,2]. Transmission of *L. monocytogenes* typically occurs through consumption of contaminated foodstuffs where ready-to-eat (RTE) foods are often compromised [3].

As RTE foods are not subjected to further control measures post processing/pre consumption food manufacturers adopt the use of modified atmosphere packaging (MAP) in an attempt to control the growth of pathogenic or spoilage organisms present in RTE foodstuffs [4]. The application of MAP utilises oxygen (O_2_), nitrogen (N_2_) and carbon dioxide (CO_2_) in an attempt to inhibit the growth of micro-organisms by decreasing O_2_ availability and increasing CO_2_ levels within food packaging [5].

Several RTE products have been implicated in listeriosis outbreaks, including dairy products, vegetables, meat and poultry products; many of which are packaged under modified atmosphere [3]. One such outbreak was caused by *L. monocytogenes* J0161 in 2000, where 30 cases of listeriosis were linked to the consumption of RTE deli turkey [6], often stored under MAP.

MAP success in pathogen control is dependent upon several factors including micro-organisms present, CO_2_ concentration, storage temperature and food type [7]. While MAP has proven success in controlling the growth of strictly aerobic micro-organisms, studies have found that the growth and survival of facultative anaerobes, such as *L. monocytogenes*, may be enhanced by its application [7]. Additionally, Francis *et al*. [7] demonstrated that the glutamate decarboxylase system, a key acid-tolerance response (ATR) component, was important for listerial survival in MAP products. Given the importance of low pH in the human immune system, conditions inducing an ATR may impact virulence [8]. Therefore, it was the aim of this study to assess the extent by which oxygen limiting conditions induce the ATR and influence simulated gastro-intestinal survival in an outbreak strain of *L. monocytogenes* J0161.

Cultures of *L. monocytogenes* strain J0161 were grown to mid-exponential (OD_600_ 0.3 – 0.5) or stationary phase in Brain Heart Infusion (BHI) broth under aerobic or anaerobic conditions. Anaerobic conditions were achieved using an anaerobic cabinet (Don Whitley, Yorkshire, UK) and monitored using anaerobic indicator strips (Oxoid, UK). For acid tolerance assays exponential phase cells were directly subjected to 5 M HCl (volume sufficient to reduce the medium to pH 3) or 2% organic acids (citric, acetic, lactic acid) in BHI. At intervals samples were removed, diluted in buffered peptone water (BPW) and plated onto BHI agar using a spiral plater (Don Whitley, Yorkshire, UK). Plates were incubated for 24–48 hours under aerobic conditions and log reductions calculated relative to time-point zero.

To model gastro-intestinal transit, SGJ was prepared as previously described [9] and adjusted to pH 1.7 or 2.5. Cells were assessed for their capacity to survive ‘short’ and ‘long’ gastric transit as described by Barbosa *et al*. [10]. Sixteen ml of pH adjusted SGJ was added to 4 ml of culture and incubated at 37°C for 60 minutes (short transit) or 120 minutes (long transit). After this time SGJ was neutralised to approximately pH 7.2 by addition of NaOH and 0.1x total volume of a 3% w/v bile salt solution, giving a final concentration of 0.3% w/v (as found *in vivo*). Following addition of bile salts cells were incubated for a further 60 minutes (short transit) or 120 minutes (long transit). Throughout simulated gastric transit, samples were removed at 30 minute intervals, dilutions made in BPW, plated using a spiral plater and incubated for 24–48 hours at 37°C. All experiments included growth controls (PBS) subjected to PBS throughout sampling, neutralised simulated gastric juice controls (SGJ) subjected to SGJ during the first phase of simulated gastric transit and bile salt only controls (bile) subjected to PBS during the first phase of simulated gastric transit followed by 0.3% w/v bile salts.

**Figure 1:**
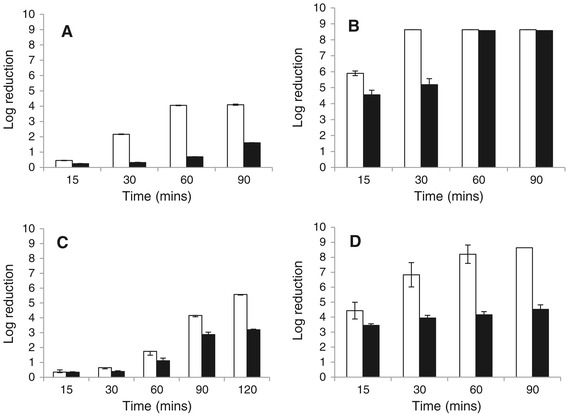
**Acid tolerance assays.** Cells were grown to mid-exponential phase at 37°C under aerobic (□) or anaerobic (■) conditions and subjected to **(A)** citric acid, 2% (w/v); **(B)** lactic acid, 2% (v/v); **(C)** acetic acid, 2% (v/v); **(D)** 5 M HCl (volume sufficient for pH 3) in BHI broth. At intervals, samples were diluted in BPW and were plated by spiral plating, log reductions were calculated relative to time-point zero after 24–48 hours incubation at 37°C. Data presents the mean ± SEOM of three biological replicates conducted in duplicate.

Anaerobic pre-conditioning significantly enhanced acid tolerance of exponential phase cells to organic and inorganic acids (*p* < 0.05) compared to those which had been grown under aerobically (*p* < 0.05). Figure 1A-D show the log reductions occurring in cultures subjected to various acids tested.

Anaerobiosis induced acid tolerance occurred during exposure to both organic and inorganic acids (Figure 1). Previously published literature has reported increased expression of glutamate decarboxylase (GAD) during anaerobic growth in *L. monocytogenes*. Given the well-established role of GAD in acid tolerance it is possible that GAD induction through anaerobic growth may initiate an acid tolerance response. This hypothesis is supported by the findings presented in this study where cells grown under anaerobic conditions displayed increased resistance to all acid types tested compared to their aerobically grown counterparts.

To establish systemic infection *L. monocytogenes* cells must overcome several barriers before reaching the intestinal epithelial cells for adhesion, invasion and subsequent infection. The first of these is survival within acidic gastric juice. The pH of the human stomach varies from pH 1–3 in healthy individuals to pH 3.5-4.5 in susceptible populations, such as the elderly, following food consumption [9], a pH range known to initiate an ATR in *L. monocytogenes* [11]. After the stomach, cells must survive exposure to the 1 litre of bile secreted daily by the liver if they are to establish systemic infection [12]. Therefore, it was deemed relevant to assess the extent by which the anaerobiosis induced ATR permitted survival of *L. monocytogenes* J0161 cells during simulated gastric transit at pH 2.5 and at pH 3.5, how this transit is impacted by growth phase, and the effect of ATR has on bile tolerance in *L. monocytogenes* J0161.

**Figure 2:**
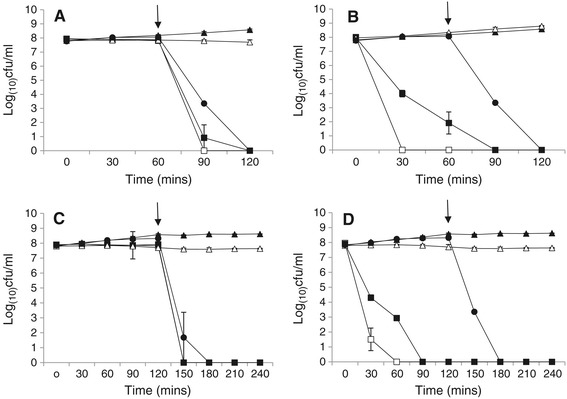
**Simulated gastro-intestinal transit of exponential phase cells.** Cells were grown to mid-exponential phase at 37°C under aerobic () or anaerobic () conditions before being subjected to ‘short’ **(A, B)** or ‘long’ **(C, D)** simulated gastric transit. For ‘short’ simulated gastric transit 4 ml of growing culture was added to 16 ml simulated gastric juice at pH 2.5 **(A)** and pH 1.7 **(B)**, for final pH values of 2.5 and 3.5 respectively. Suspensions were incubated at 37°C for 60 minutes and then neutralised to ~ pH 7.2 and 0.1x volumes of 3% (w/v) bile salt solution added (giving a final bile salt concentration of 0.3% w/v, as seen *in vivo*). Suspensions were incubated for a further 60 minutes at 37°C. For ‘long’ simulated gastric transit 4 ml of growing culture was added to 16 ml simulated gastric juice at pH 2.5 **(C)** and pH 1.7 **(D)**, for final pH values of 2.5 and 3.5 respectively. Suspensions were incubated at 37°C for 120 minutes and then neutralised to ~ pH 7.2 and 0.1x volumes of 3% (w/v) bile salt solution added (giving a final bile salt concentration of 0.3% w/v, as seen *in vivo*). Suspensions were incubated for a further 120 minutes at 37°C. Throughout testing, samples were taken at half hourly intervals, diluted in BPW and spiral plated on BHI agar. Plates were enumerated after 24–48 hours incubation at 37°C. PBS (), SGJ () and bile () controls were also included. Limit of detection = 3x10^2^cfu ml^−1^. Arrows indicate pH neutralization and addition of bile salts. Data is representative of three independent experiments conducted in duplicate.

When challenged by SGJ at pH 3.5 *L. monocytogenes* J0161 cells remained viable. There were no differences in viable counts between those subjected to SGJ and control cells (*p* > 0.05), therefore pre-conditioning under oxygen limiting conditions had no effect on resistance to SGJ at pH 3.5 (*p* > 0.05) (Figure 2A and C). However, there was evidence of anaerobiosis induced acid tolerance when cells were subjected to SGJ at pH 2.5. When grown under oxygen limiting conditions, *L. monocytogenes* cells remained viable for 60 minutes following SGJ exposure. During both short and long simulated gastric transit, the number of recoverable cells was significantly greater following anaerobic growth compared to those grown aerobically (*p* < 0.05) (Figure 2B and D). While this demonstrates the potential for anaerobiosis induced acid tolerance to permit successful passage of *L. monocytogenes* through the low pH of the stomach, it should be noted that exponential phase cells were unable to survive bile salt exposure, irrespective of growth conditions. Bile is a potent antimicrobial which has been previously reported to rapidly inactivate exponential phase cells at physiological concentrations [13], growth under oxygen limiting conditions did not induce a cross protection against such exposure.

**Figure 3:**
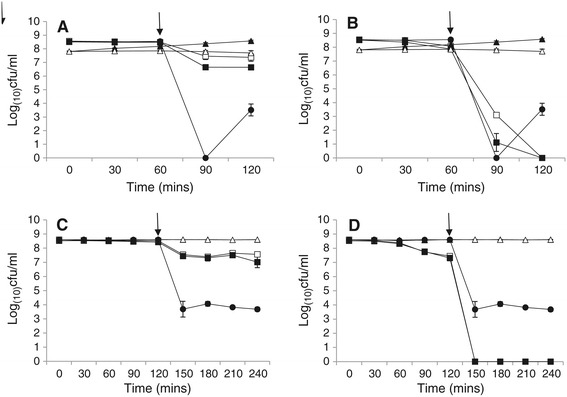
**Simulated gastro-intestinal transit of stationary phase cells.** Cells were grown to stationary phase at 37°C under aerobic () or anaerobic () conditions before being subjected to ‘short’ **(A, B)** or ‘long’ **(C, D)** simulated gastric transit. For ‘short’ simulated gastric transit 4 ml of growing culture was added to 16 ml simulated gastric juice at pH 2.5 **(A)** and pH 1.7 **(B)**, for final pH values of 2.5 and 3.5 respectively. Suspensions were incubated at 37°C for 60 minutes, neutralised to ~ pH 7.2 and 0.1x volumes of 3% (w/v) bile salt solution added (giving a final bile salt concentration of 0.3% w/v, as seen *in vivo*). Suspensions were incubated for a further 60 minutes at 37°C. For ‘long’ simulated gastric transit 4 ml of growing culture was added to 16 ml simulated gastric juice at pH 2.5 **(C)** and pH 1.7 **(D)**, for final pH values of 2.5 and 3.5 respectively. Suspensions were incubated at 37°C for 120 minutes, neutralised to ~ pH 7.2 and 0.1x volumes of 3% (w/v) bile salt solution added (giving a final bile salt concentration of 0.3% w/v, as seen *in vivo*). Suspensions were incubated for a further 120 minutes at 37°C. Throughout testing, samples were taken at half hourly intervals, diluted in BPW and spiral plated on BHI agar. Plates were enumerated after 24–48 hours incubation at 37°C. PBS (), SGJ () and bile () controls were also included. Limit of detection = 3x10^2^cfu ml^−1^. Arrows indicate pH neutralization and addition of bile salts. Data is representative of three independent experiments conducted in duplicate.

While exponential phase cells were unable to survive simulated gastric transit, stationary phase cells were able to survive transit through our model system in certain situations (Figure 3A and C). This was a relevant finding given that bacterial cells often exist in a stationary phase state [14]. Upon entry into stationary phase, the resistance properties of cells is altered [15-17], resulting in increased resistance to several antimicrobials, including acids [15-17] and bile [13]. This was seen in our study where stationary phase cells readily overcame SGJ at pH 2.5 (Figure 3B and D), representing a potential risk even to those who are not deemed ‘pre-disposed’ through high gastric pH. However, following such exposure, cells were unable to survive bile salt exposure beyond 30 minutes. Aerobically grown cells demonstrated enhanced tolerance to 0.3% bile salts but this was insufficient to permit viable cells at the end of ‘transit’ in our system.

As with exponential phase cells, those grown to stationary phase were readily able to survive exposure to pH 3.5 SGJ (Figure 3A and C). However, they were able to survive bile salt exposure. Interestingly, mild acid stress of stationary phase cells induced a cross protection to bile salt relative to bile control cells. Pre-exposure to pH 3.5 SGJ resulted in significantly greater recovery of cells during the ‘intestinal’ phase of transit (*p* < 0.05). The extent of this cross protection was pre-condition dependent, those grown aerobically possessing greater bile salt tolerance than those grown anaerobically (*p* < 0.05), suggesting a possible link between acid adaptation and *Listeria* persistence during gastric transit.

In summary, this report demonstrates the induction of an anaerobiosis induced ATR in *L. monocytogenes* J0161. When applying these findings to a novel simulated gastro-intestinal transit model it appears that this response may aid transit of *L. monocytogenes* through the low pH of the stomach. Previous studies have investigated the effects of oxygen limitation on *L. monocytogenes* virulence [18,19]. In these studies oxygen limitation was found to enhance invasive and infective potentials respectively. While this has been largely attributed to increased expression and secretion of adhesion proteins [18], here we provide evidence to suggest that oxygen limitation enhances resistance to gastric juice, thus increasing the likelihood of invasive infection [19]. Furthermore, cells were found to possess growth phase dependent bile tolerance which was impacted by pre-exposure to low pH; a finding which may explain the ability of *L. monocytogenes* to persist in the presence of bile *in vivo*.

While this study was carried out using a single strain, the isolate originated from a human listeriosis case which was part of a large-scale outbreak culminating in 29 human infections and four deaths [20] and so can be considered an important model organism when investigating *Listeria* pathogenesis.

## Abbreviations

SGJ: Simulated gastric juice
ATR: Acid tolerance response
BPW: Buffered peptone water
GAD: Glutamate decarboxylase
PBS: Phosphate buffered saline

## References

[1] Garner MR, Njaa BL, Wiedmann M, Boor KJ (2006). Sigma B contributes to *Listeria monocytogenes* gastrointestinal infection but not to systemic spread in the guinea pig infection model. Infect Immun.

[2] Allerberger F, Wagner M (2010). Listeriosis: a resurgent foodborne infection. Clin Microbiol Infect.

[3] Cartwright EJ, Jackson KA, Johnson SD, Graves LM, Silk BJ, Mahon BE. Listeriosis outbreaks and associated food vehicles, United States, 1998–2008. Emerging Infectious Diseases 2013 [Online] [Accessed 02.11.14] available from: http://dx.doi.org/10.3201/eid1901.120393. 10.3201/eid1901.120393 PMC3557980 23260661

[4] Phillips CA (1996). Review: modified atmosphere packaging and its effects on the microbiological quality and safety of produce. Int J Food Sci Technol.

[5] Lungu B, Ricke SC, Johnson MG (2009). Growth, survival, proliferation and pathogenesis of *Listeria monocytogenes* under low oxygen or anaerobic conditions: A review. Anaerobe.

[6] Olsen SJ, Patrick M, Hunter SB, Reddy V, Kornstein L, MacKenzie WR (2005). Multistate outbreak of *Listeria monocytogenes* infection linked to delicatessen turkey meat. Clin Infect Dis.

[7] Francis GA, Scollard J, Meally A, Bolton DJ, Gahan CGM, Cotter PD (2007). The glutamate decarboxylase acid resistance mechanism affects survival of *Listeria monocytogenes* LO28 in modified atmosphere‐packaged foods. J Appl Microbiol.

[8] Gahan CGM, Hill C. Listeria monocytogenes: Survival and adaptation in the gastrointestinal tract. Front Cell Infect Microbiol. 2014. doi:10.3389/fcimb.2014.00009. 10.3389/fcimb.2014.00009 PMC3913888 24551601

[9] Ivy RA, Wiedmann M, Boor KJ (2012). *Listeria monocytogenes* grown at 7°C shows reduced acid survival and an altered transcriptional response to acid shock compared to *L. monocytogenes* grown at 37°C. Appl Environ Microbiol.

[10] Barbosa J, Borges S, Magalhaes R, Ferreira V, Santos I, Silva J (2012). Behaviour of *Listeria monocytogenes* isolates through gastro-intestinal tract passage simulation, before and after two sub-lethal stresses. Food Microbiol.

[11] Koutsoumanis KP, Kendall PA, Sofos JN (2003). Effect of food processing-related stresses on acid tolerance of *Listeria monocytogenes*. Appl Environ Microbiol.

[12] Hofmann AF, Arias IM, Boyer JL, Fausto N, Jackoby WB, Schachter DA, Shafritz DA (1994). Bile acids. The liver: biology and pathobiology.

[13] Begley M, Gahan CGM, Hill C (2002). Bile stress response in *Listeria monocytogenes* LO28: adaptation, cross-protection, and identification of genetic loci involved in bile resistance. Appl Environ Microbiol.

[14] Kolter R, Siegele DA, Tormo A (1993). The stationary phase of the bacterial life cycle. Ann Rev Microbiol.

[15] Arnold KW, Kaspar CW (1995). Starvation-and stationary-phase-induced acid tolerance in *Escherichia coli* O157: H7. Appl Environ Microbiol.

[16] Benjamin MM, Datta AR (1995). Acid tolerance of enterohemorrhagic *Escherichia coli*. Appl Environ Microbiol.

[17] King T, Lucchini S, Hinton JC, Gobius K (2010). Transcriptomic analysis of *Escherichia coli* O157: H7 and K-12 cultures exposed to inorganic and organic acids in stationary phase reveals acidulant-and strain-specific acid tolerance responses. Appl Environ Microbiol.

[18] Burkholder KM, Kim KP, Mishra KK, Medina S, Hahm BK, Kim H (2009). Expression of LAP, a SecA2-dependent secretory protein, is induced under anaerobic environment. Microbes Infect.

[19] Andersen JB, Roldgaard BB, Christensen BB, Licht TR (2007). Oxygen restriction increases the infective potential of *Listeria monocytogenes in vitro* in Caco-2 cells and *in vivo* in guinea pigs. BMC Microbiol.

[20] Orsi RH, Borowsky ML, Lauer P, Young SK, Nusbaum C, Galagan JE (2008). Short-term genome evolution of *Listeria monocytogenes* in a non-controlled environment. BMC Genomics.

